# Psychotherapeutische Interventionen bei Burnout – Ein Umbrella-Review und Impulse für die Therapie

**DOI:** 10.1007/s00103-024-03961-y

**Published:** 2024-10-15

**Authors:** Sarah Kern, Lucia Jerg-Bretzke, Petra Beschoner

**Affiliations:** 1 Klinik für Psychosomatische Medizin und Psychotherapie, Universitätsklinikum Ulm, Ulm, Deutschland; 2 IB Hochschule für Gesundheit und Soziales, Stuttgart, Deutschland; 3 Akutklinik Bad Saulgau, Deutschland; 4 Klinik für Psychosomatische Medizin und Psychotherapie, Sektion Medizinische Psychologie, Universitätsklinikum Ulm, Albert-Einstein-Allee 7, 89081 Ulm, Deutschland

**Keywords:** Burnout, Umbrella-Review, Therapie, Ressourcen, Behandlungsansätze, Therapy, Burnout, Umbrella review, Resources, Interventions

## Abstract

**Hintergrund:**

Burnout stellt keine eigenständige medizinische Diagnose nach ICD-11 dar, jedoch leiden laut Studienlage viele Menschen daran. Dies hat auch erhebliche volkswirtschaftliche und gesundheitspolitische Konsequenzen, da Burnout oftmals mit Arbeitsunfähigkeit und erheblichen Folgeerkrankungen einhergeht. Diese Arbeit soll einen Überblick über bereits angewandte und evaluierte Therapieansätze für Burnout vorstellen und zusätzlich aufzeigen, in welchem Bereich vielversprechende, jedoch noch wenig erforschte therapeutische Unterstützungsmöglichkeiten für Betroffene liegen.

**Methoden:**

Systematische Literaturrecherche von 2010 bis 2024 in PubMed und Google Scholar für ein Umbrella-Review. Suchbegriffe waren: „burnout“, „therapy“ und „intervention“, kombiniert mit „systematic review“, „meta-analysis.“ Insgesamt 10 systematische Reviews bzw. Metaanalysen entsprachen den Einschlusskriterien.

**Ergebnisse:**

In 5 Reviews bzw. Metaanalysen wurde bei den untersuchten Therapieansätzen eine spezielle Berufsgruppe fokussiert (Pflegefachkräfte, Ärzte und Lehrer), die anderen bezogen sich auf Studenten oder verschiedene Personengruppen. In 7 Arbeiten wurden die Effekte von Achtsamkeitstraining auf Burnout gezeigt. Für kognitive Verhaltenstherapie (KVT) wurde Effekte in 4 Arbeiten nachgewiesen. Als wirksam erwiesen sich auch die Akzeptanz- und Commitment-Therapie (ACT) sowie die Rational-Emotive-Therapie (RET).

**Diskussion:**

Es muss noch erforscht werden, ob etablierte Gedanken- und Verhaltensmuster durch die genannten Ansätze der Burnout-Therapie auch langfristig verändert werden. Der berufliche Kontext sollte mehr Beachtung finden. Hilfreich erscheinen hier ressourcenorientierte Ansätze, die Aspekte wie Optimismus, Kontrollerleben, Selbstwirksamkeit und Selbstakzeptanz einbeziehen.

**Zusatzmaterial online:**

Zusätzliche Informationen sind in der Online-Version dieses Artikels (10.1007/s00103-024-03961-y) enthalten.

## Abstract (en)

**Background:**

Although burnout is not an F‑Diagnosis in the ICD-11, many people are suffering from it. This has many economic and health-related consequences, because burnout is closely tied to an inability to work and significant secondary illnesses. This article provides an umbrella review of therapy approaches for burnout that have already been used and evaluated and shows in which areas there are promising, but still little researched, therapeutic support options for those affected.

**Methods:**

A systematic literature search of studies was carried out in PubMed and Google Scholar between 2010 and 2024. The search terms were “burnout,” “therapy,” and “intervention” combined with “systematic review” and “meta-analysis.” Overall, 10 systematic reviews or meta-analyses met the inclusion criteria.

**Results:**

In five systematic reviews or meta-analyses, the therapy approaches focused on a specific professional group (nurses, doctors, and teachers); the others related to students or various groups of people. In seven studies, the effects of mindfulness-based stress reduction on burnout were shown. The effects of cognitive behavioral therapy (CBT) were shown in four studies. Acceptance and commitment-therapy (ACT) as well as rational emotive therapy (RET) also proved to be effective.

**Conclusion:**

It still needs to be researched whether established thinking and behavioral patterns can be changed through the therapies discussed. The professional context should be given more attention. Some resource-oriented approaches like optimism, a sense of control, self-efficacy, and self-acceptance seem promising in the treatment of burnout.

## Einleitung

Herausfordernde Arbeitsbedingungen, Leistungserbringung bis zur Erschöpfung, gepaart mit fehlendem Ausgleich (Work-Life-Balance) führen bei Beschäftigten immer häufiger zu einem Zustand des „Ausgebranntseins“ (Burnout). Dieser kann zu Folgestörungen, wie zum Beispiel einer Depression, führen, wodurch sich auch längere Zeiträume von Arbeitsunfähigkeit ergeben können. Bis heute liegt keine allgemeingültige Definition für Burnout vor, es gibt jedoch Konsens darüber, dass es sich um einen schleichend einsetzenden Prozess handelt, bei dem ein Zustand physischer, kognitiver und emotionaler Erschöpfung eintritt, verbunden mit Motivationsverlust, Unlust, Zynismus, Verbitterung und Desillusionierung, der oft geprägt ist von einer Entfremdung von sich selbst und von anderen [1].

Die international am meisten etablierte Definition für Burnout stammt von der Sozialpsychologin Christina Maslach und ihren Kollegen, die ein dreidimensionales Modell auf faktorenanalytischer Basis entwickelten [1]. Sie betrachten Burnout gemäß ihrem Maslach Burnout Inventory (MBI) als ein Syndrom, das sich aus den Dimensionen „emotionale Erschöpfung“, „Depersonalisation“ (Zynismus) und „verminderte Leistungsfähigkeit“ zusammensetzt [2]. Emotionale Erschöpfung ist dabei die Hauptkomponente, durch die sich Burnout entwickelt. Sie manifestiert sich am deutlichsten [3] und wirkt schließlich auch als Schlüsselfaktor hinsichtlich negativer Konsequenzen [2]. Sie beschreibt die emotionale Überforderung im beruflichen Kontakt mit Menschen. Die Betroffenen bringen nicht mehr die nötige Empathie auf, es entsteht das Gefühl, ausgelaugt zu sein. Die zweite Dimension Depersonalisation beschreibt eine zynische und ablehnende Wahrnehmung anderer, was in Gleichgültigkeit oder abwertenden Äußerungen und Verhaltensweisen deutlich wird. Unter reduzierter Leistungsfähigkeit versteht man ein Gefühl von abnehmender Kompetenz verbunden mit einem zunehmend negativen Selbstbild und Insuffizienzerleben im Beruf. Im Folgenden werden Symptome, Auswirkungen, Diagnose, Risikogruppen und Ursachen von Burnout kurz beschrieben.

Burnout ist mit höherem physiologischen Disstress sowie erhöhter Erregbarkeit assoziiert und kann so beispielsweise zu Schlafstörungen, Spannungszuständen und psychosomatischen Beschwerden führen [4]. Bei anhaltender Belastung und Fortschreiten des Burnout-Syndroms können Ängste, Substanzgebrauch und psychische Syndrome bis hin zu manifesten psychischen Erkrankungen auftreten [4]. Weiter können auf der organisationalen Ebene eine niedrige Arbeitszufriedenheit sowie Präsentismus und Absentismus auftreten [5]. Insgesamt sind die Leistungsfähigkeit und die Produktivität bei Burnout reduziert [6].

Als Hauptrisikogruppen lassen sich helfende und beratende Berufsgruppen identifizieren [7], die viel Kontakt mit Menschen haben (z. B. Lehrer, Ärzte, Gesundheitsberufe). Auch wenn zunächst hauptsächlich diese Gruppen im Fokus standen, ist Burnout ein Konstrukt aus der Arbeits- und Organisationspsychologie, das sich auf alle Berufsgruppen bezieht. Burnout stellt daher im eigentlichen klinischen Sinn keine medizinische Diagnose dar. In der 10. Version der Internationalen Klassifikation der Krankheiten (ICD-10) wurde Burnout als psychische Störung mit dem Code Z73.0 unter „Probleme mit Bezug auf Schwierigkeiten bei der Lebensbewältigung“ aufgeführt und im ICD-11 unter „Problematik in Verbindung mit Berufstätigkeit oder Arbeitslosigkeit“ (Code QD 85).

Aus medizinisch-therapeutischer Sicht sollte Burnout als ein ätiologisch relevanter Faktor für die Entwicklung und Aufrechterhaltung manifester psychischer Störungen betrachtet werden. Entsprechend könnte eine gezielte Intervention das Fortschreiten eines Burnout-Syndroms und die mögliche Entwicklung von Folgeerkrankungen verhindern. Die Diagnose eines Burnouts kann entstigmatisierend wirken und die Hemmschwelle senken, sich bei psychischen Belastungen Hilfe zu suchen.

In der Abgrenzung zu weiteren psychischen Diagnosen birgt Burnout in seiner Konzeption Spezifika, die sich nicht allein durch die Diagnosen Depression, Anpassungsstörung oder Angststörung erklären lassen. Beim Burnout liegt isoliert eine emotionale Erschöpfung vor, die bei einer Depression oder Anpassungsstörung begleitet wird von körperlicher Erschöpfung und Kraftlosigkeit. Diese körperliche Erschöpfungskomponente fehlt beim Burnout. Die Dimension des Zynismus kann im Allgemeinen im Rahmen einer Depression, Anpassungsstörung oder Angststörung nicht beobachtet werden, im Gegenteil erleben sich depressive oder ängstliche Patienten häufig besonders durchlässig und leiden mit anderen Betroffenen mit.

Hinsichtlich einer Ursachenzuweisung lassen sich individuenzentrierte, arbeits- und organisationspsychologische sowie sozialpsychologische Ansätze unterscheiden. Dabei spielt die Arbeit im Sinne der Arbeitsbedingungen, der Arbeitsmenge, des Zeitdrucks oder psychosozialer Arbeitsbelastungen eine besondere Rolle [8]. Nach dem sozialpsychologischen Ansatz liegt die Ursache von Burnout v. a. im Verlust von moralischen Zielsetzungen. Die Arbeitsstressoren führen demnach nur zum Burnout, wenn der individuelle subjektive Bedeutungsrahmen der eigenen Arbeit nicht mehr sinnhaft ideologisch zugeordnet werden kann [9]. Der individuenzentrierte Ansatz bezieht sich auf personenbezogene Merkmale als Ursache des Burnouts, so werden bspw. die Enttäuschung und Frustration infolge unrealistischer beruflicher Zielsetzungen genannt. Freudenberger identifizierte bereits 1974 für besonders engagierte, idealistische Menschen ein hohes Risiko, auszubrennen. Der arbeits- und organisationspsychologische Ansatz nennt spezielle arbeitsbezogene Determinanten wie Rollenstress, zu wenig Autonomie, mangelnde soziale Unterstützung sowie geringe Entscheidungsspielräume als ursächlich für ein hohes Burnout-Risiko [10].

Nach dem von Siegrist formulierten Modell der beruflichen Gratifikationskrisen ist die Balance zwischen den Anforderungen im Beruf und den dafür erhaltenen Wertschätzungen wie Gehalt, Anerkennung oder Selbstwertsteigerung nicht mehr gegeben [11] und dies steht im Zusammenhang mit der Entwicklung stressassoziierter Folgestörungen wie Burnout.

Dass Burnout keine medizinische Diagnose im Sinne der Klassifikationssysteme ist und in seiner Konzeption als eine Art „Arbeitsstörung“ verstanden wird, hat zur Folge, dass Burnout zwar relativ intensiv in der Arbeits- und Organisationspsychologie erforscht wird [12], aber kaum im Fokus der Psychotherapieforschenden steht. Gleichzeitig geht Burnout aber, wie bereits beschrieben, mit erheblichen Symptomen einher, die bei den Betroffenen zu persönlichem Leiden, zur Entwicklung einer „echten“ psychischen oder körperlichen Erkrankung [2] wie auch zu einer Einschränkung der Produktivität führen können [2]. Hier wird deutlich, wie wichtig frühe therapeutische Interventionen sind, um Burnout zu adressieren, bevor sich beispielsweise eine Depression daraus entwickelt. Aber auch bei bereits bestehender „Folgeerkrankung“, wie beispielsweise Depression, ist die fokussierte therapeutische Adressierung des Burnouts als auslösender Faktor sinnvoll.

Laut AOK-Report haben sich die Arbeitsunfähigkeitstage (AU) aufgrund einer Z73.0-Diagnose zwischen 2012 und 2021 um mehr als 50 % erhöht. Auch an diesen Zahlen zeigt sich, dass die Regeneration bei Burnout zeit- und kostenintensiv ist [13]. Neben den genannten Ausfallzeiten hat Burnout insbesondere hinsichtlich der Arbeitsqualität in den helfenden und beratenden Tätigkeiten Konsequenzen: So ist zum Beispiel bei Ärzten Burnout mit niedrigerer Jobzufriedenheit verbunden und das Risiko für Fehler im Umgang mit den Patienten doppelt erhöht [14].

Ziel dieser Übersichtsarbeit ist es, auf Basis einer systematischen Literaturrecherche zu evaluierten Therapieansätzen bei Burnout ein Bewusstsein für die Spezifika des Burnouts zu schaffen und ein therapeutisches Vorgehen herauszustellen, das Betroffenen früh zielgerichtet helfen kann, bevor sie eine schwerwiegende Störung entwickeln.

## Methoden

Um eine große inhaltliche Spannbreite zu erreichen, wurde das Format eines Umbrella-Reviews gewählt – eine breitgefächerte Übersichtsarbeit über systematische Reviews und/oder Metaanalysen.

**Abb. 1:**
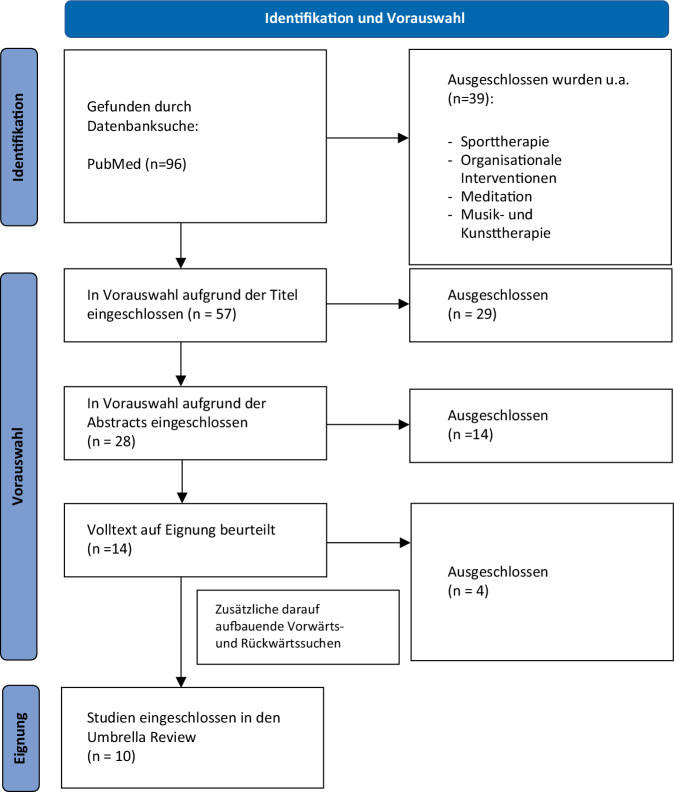
Studien-Screening-Prozess. PRISMA-Flow Diagramm ([15]; eigene Abbildung)

Die systematische Literatursuche erfolgte unter Einhaltung der PRISMA-Statements [15] im Zeitraum vom 01.02.2024 bis zum 03.05.2024 über die Datenbanken PubMed und Google Scholar. Ein Protokoll nach den PRISMA-Richtlinien wurde parallel angefertigt (siehe Onlinematerial) und bei OSF-Registries (osf.io/xt7pa/) registriert. Wir haben uns auf die Jahre 2010–2024 konzentriert, um möglichst aktuelle Reviews und Metaanalysen zu identifizieren. Für die Suche wurden die Begriffe „burnout“, „therapy“, kombiniert mit „systematic review“, „meta-analysis“ sowie „intervention“ verwendet. Es wurden nur Studien mit aufgenommen, die diese Suchbegriffe im Titel oder im Abstract haben. Duplikate wurden in einem zweiten Schritt entfernt. Die restlichen Publikationen wurden mit Blick auf inhaltliche Relevanz anhand der Titel, dann der Abstracts und schließlich des gesamten Textes überprüft. Sport‑, Kunst- oder ähnliche Therapieformen wurden ausgeschlossen, da sie nicht im engeren Sinne zu Psychotherapie gehören. In Abb. 1 wird das Rechercheverfahren anhand eines PRISMA-Flow-Diagramms dargestellt. Insgesamt 10 systematische Reviews bzw. Metaanalysen entsprachen den Einschlusskriterien.

## Ergebnisse

In den verschiedenen Studien wurden mehrfach die Effekte von Achtsamkeitstraining auf Burnout gezeigt: Eine detaillierte Übersicht über die Hauptergebnisse gibt Tab. 1. In 7 der 10 analysierten Metaanalysen bzw. systematischen Reviews wurden die Effekte von Achtsamkeitstraining auf Burnout bestätigt [16–22]. In einem Review wurden Daten von insgesamt 623 Pflegefachkräften [16] aus 17 Artikeln zusammengefasst, in einem anderen Daten von 1077 Pflegefachkräften aus 14 Reviews [17]. In beiden wurde der Effekt von Achtsamkeitstraining auf die verschiedenen Facetten von Burnout gezeigt. In den anderen genannten Studien wurden die Effekte auch für andere Berufsgruppen belegt.

**Tab. 1:** Übersicht der Reviews bzw. Metaanalysen zu Therapieansätzen bei Burnout

Studie [Referenznr.]	Typ (Anzahl der Studien)	Personengruppe	Wirksame Interventionen	Hauptpunkte
*Suleiman-Martos et al. 2020* [16]	Review und Metaanalyse (17)	Pflegefachkräfte	Achtsamkeitstraining	Die Dauer der Trainings umfasst zwischen 3 und 6 Wochen. Die Studien zeigen, dass die Teilnahme an achtsamkeitsbasierten Trainings zu einer Reduktion der emotionalen Erschöpfung und der Depersonalisierung sowie zu einer Erhöhung der persönlichen Leistungsfähigkeit der Pflegekräfte führt
				Einzelne Studien zeigten, dass die positiven Effekte über 6 Monate erhalten blieben, andere, dass nur die Dimension der emotionalen Erschöpfung über einen Zeitraum von 3 Monaten nach der Intervention erhalten blieb
*Luken und Sammons 2018* [18]	Systematisches Review (8)	Verschiedene	Achtsamkeitstraining	Die Achtsamkeitspraxis reduziert Burnout bei Gesundheitsfachkräften und Lehrern, zudem zeigten sich signifikant weniger emotionale Erschöpfung und ein größeres Gefühl persönlicher Leistung
*Tement et al. 2021* [19]	Systematisches Review (18)	Ärzte	Achtsamkeitstraining	MBSR hat positive Auswirkungen auf Empathie, Wohlbefinden und die Reduktion von Burnout bei Ärzten und trägt zu einer verbesserten Lebensqualität bei
				Die Interventionen haben langfristige Effekte: 3–6 Monate nach Abschluss halten die Effekte noch an
				Einen positiven Effekt hat auch der Peer-Support. Durch Austausch kann das Gefühl der Isolation reduziert werden und das allgemeine Wohlbefinden gefördert werden. Die Achtsamkeitsfähigkeiten verbesserten darüber hinaus die Fähigkeit, aufmerksamer Patienten gegenüber zu sein und effektiver zu reagieren
*Iancu et al. 2017* [20]	Metaanalyse (23)	Lehrer	CBT und Achtsamkeitstraining	Interventionen zur Reduzierung von Burnout haben kleine Effektstärken, insbesondere bei der emotionalen Erschöpfung und der Leistungsfähigkeit, jedoch keine bei Depersonalisation
*Jaworska-Burzynska et al. 2016* [21]	Systematisches Review (22)	Verschiedene	CBT und Achtsamkeitstraining	CBT wurde in mehreren Studien als wirksam bei der Reduktion von Burnout-Symptomen beschrieben
				Bei MBSR zeigten sich die Ergebnisse weniger eineindeutig
*Korczak et al. 2012* [23]	Systematisches Review (17)	Verschiedene	CBT	Der Einsatz kognitiver Verhaltenstherapie (CBT) führt in der Mehrheit der Studien zu Verbesserungen der emotionalen Erschöpfung
*Madigan et al. 2023* [22]	Systematisches Review und Metaanalyse (17)	Studenten	CBT, Achtsamkeit und RET	Interventionen zur Reduktion von Burnout bei Schülern und Studenten sind insgesamt wirksam. Der aggregierte Effekt zeigt, dass verschiedene Ansätze, wie Achtsamkeit, RET und CBT signifikante Verbesserungen in der Reduktion von Burnout-Symptomen bewirken
				Die größten Effektstärken liegen für die RET vor
				Die Studienergebnisse können mit großer Sicherheit auf Studenten mit einer hohen Burnout-Belastung übertragen werden; fraglich ist, ob die Ergebnisse auch für moderate Burnout-Symptome gelten können
*Towey-Swift et al. 2023* [24]	Systematisches Review (9)	Verschiedene	ACT	In 11 von 14 Studien zeigen ACT-Interventionen positive Effekte bzgl. Verringerung von Burnout
				ACT-Interventionen umfassten zwischen 1 und 8 Sitzungen mit einer Gesamtdauer von 4,5–16 h
				Die größten Effekte traten bei der Durchführung von individuellen Einzelsitzungen auf
				ACT so wirksam wie CBT
*Ramachandran et al. 2023* [17]	Systematisches Review und Metaanalyse (14)	Pflegefachkräfte	Achtsamkeitstraining	MBSR kann Burnout-Symptome signifikant reduzieren, wobei eine signifikante Verringerung der emotionalen Erschöpfung und eine Verbesserung der persönlichen Leistung (nicht aber eine Verringerung der Depersonalisierung) erreicht wird
				Im Vergleich zu aktiven Vergleichsstudien erwies sich MBSR auch bei der Verringerung von psychischem Stress als wirksamer und zeigte einen Effekt bei der Verringerung von Stress, Angst, Depression und Burnout
*Reeve et al. 2018* [25]	Systematisches Review und Metaanalyse (7)	Pflegefachkräfte	ACT	ACT-Interventionen erwiesen sich als wirksam bei der Reduzierung von psychischem Stress
				In diesem Fall zielte die ACT darauf ab, ein werteorientiertes Leben zu unterstützen
				Kein signifikanter Effekt hinsichtlich der direkten Reduzierung von Burnout

*ACT* Akzeptanz- und Commitment-Therapie, *CBT* Cognitive Based Therapy (*KVT* kognitive Verhaltenstherapie), *MBSR* Mindfulness-based Stress Reduction, Achtsamkeitstraining, *RET* Rational-Emotive-Therapie

Weiter wurde in 3 von 10 Studien die Wirksamkeit von kognitiver Verhaltenstherapie (KVT; [21–23]) bei verschiedenen Berufsgruppen nachgewiesen. Zudem wurde die Akzeptanz- und Commitment-Therapie (ACT) in 2 verschiedenen Metaanalysen [24, 25] für die Reduktion von Burnout als geeignete Form der Therapie belegt. Die Rational-Emotive-Therapie (RET), eine Form der kognitiven Verhaltenstherapie, die Grundüberzeugungen hinterfragt, erwies sich ebenfalls in einem systematischen Review mit Metaanalyse als wirksam [22].

## Diskussion

Ein Ziel des vorliegenden Artikels war es, zunächst einen Überblick über vorhandene, gut erforschte und wirksame Therapieansätze für Burnout zu geben. Dabei zeigte sich, dass es 4 verschiedene Therapiemöglichkeiten gibt, die immer wieder erfolgreich zum Einsatz kommen: Achtsamkeitstraining, Akzeptanz- und Commitment-Therapie, kognitive Verhaltenstherapie und die Rational-Emotive-Therapie. Sie haben jedoch jeweils ihre eigenen Vor- und Nachteile. Es ist wichtig, sowohl die Vorteile als auch die Grenzen jeder Therapie zu verstehen.

### Kognitive Verhaltenstherapie (KVT)

Die kognitive Verhaltenstherapie hat bei Burnout den Vorteil, dass sie sich auf konkrete Probleme und Lösungsstrategien konzentriert [27]. In einigen Studien lag der Fokus auf der Steigerung der Selbstwirksamkeit, die mittels Aktivitätspläne und der Beobachtung dysfunktionaler Gedanken sowie mit dem Einüben alternativer Gedanken und deren Umsetzung in funktionaleres Verhalten erreicht werden soll. Eine der Studien, die von Madigan [22] untersucht wurden, befasst sich mit der Psychoedukation in Bezug auf Stress und dem Erlernen eines daraus abgeleiteten veränderten Umgangs mit Sorgen und Unsicherheit, eine weitere mit dem Aufschreiben von dysfunktionalen Gedanken, um diese zu verändern [22]. In diesen beiden Studien zeigten sich reduzierte Werte bei den Burnout-Faktoren Zynismus und emotionale Erschöpfung.

Die Durchführungszeit einer KVT lag laut einem Review bei 1 Monat bis zu einem halben Jahr [21], wobei es sich um eine sehr große Spannweite handelt. In einem anderen Review erwiesen sich Therapien mit einem Zeitraum zwischen 3 und 4 Monaten mit 15–20 Sitzungen als wirksam [23]. Aus den Ergebnissen der Studien kann man schlussfolgern, dass kognitive Verhaltenstherapie als Intervention bei Burnout wirksam ist, indem sie dabei hilft, dysfunktionale Gedanken zu identifizieren und diesen funktionale Gedanken in Hinblick auf einen gesünderen Umgang mit Stress entgegenzusetzen.

Mögliche Nachteile der KVT-Ansätze der vorliegenden Studien könnten sich aus dem alleinigen Fokus auf das „Hier und Jetzt“ ergeben. Die tiefer liegenden Ursachen, wie beispielsweise die Verinnerlichung von Leistungsstreben und Einsatzbereitschaft, die sich in Glaubenssätzen, wie beispielsweise: „Du musst alles schaffen!“ oder „Du darfst keine Enttäuschung sein“, manifestieren und in Konflikten als emotionale Übertragungsmuster zeigen, können so nicht komplett erreicht werden. Bei von Burnout betroffenen Personen, die sehr leistungsorientiert sind, kann selbst das Ziel der Behandlung als Herausforderung erlebt werden, die es mit maximalem Erfolg in kürzester Zeit zu bewältigen gilt. Hier kann die Therapie jedoch nicht nachhaltig wirksam werden.

### Achtsamkeitstraining bzw. Mindfulness-based Stress Reduction (MBSR)

Das Achtsamkeitstraining bzw. speziell die Methode „Mindfulness-based Stress Reduction“ (MBSR; [26]) dienen v. a. der Stressbewältigung. Durch bewusstes Wahrnehmen und Handeln im „Hier und Jetzt“ sollen z. B. auch bewertende Gedanken reduziert werden. Burnout geht mit nahezu automatisierten Wahrnehmungsabläufen und Verhaltensmustern wie Grübeln, dysfunktionalen Gedanken, Sorgen und Ängsten sowie Zynismus und Rückzug einher. Hier kann Achtsamkeit dazu dienen, die verzerrte Wahrnehmung und ungünstige Verhaltensweisen zu erkennen und eine andere, zuträgliche Haltung und einen funktionaleren Umgang zu entwickeln. Achtsamkeitsübungen helfen den Betroffenen zu erkennen, dass der Prozess der Wahrnehmung in ihnen selbst stattfindet und die Antwort auf einen Reiz ihre eigene ist. Dies ermöglicht eine Differenzierung in der Wahrnehmung von Stress und in der Selbstwahrnehmung und ermöglicht so eine Veränderung in der Wahrnehmung und im Verhalten, die zu einer Reduktion der Burnout-Symptomatik führen kann.

Allerdings sind Achtsamkeitsübungen in den Studien nicht in einen therapeutischen Gesamtkontext eingebunden. Dazu zählen u. a. Biografiearbeit, Entwicklung eines Störungsmodells, die Fokussierung von Beziehungsaspekten im Zusammenhang mit der Burnout-Symptomatik. Hier liegt auch die Grenze der Achtsamkeitsübungen/des Achtsamkeitstrainings bei Burnout, es verschafft eine Linderung, hilft aber unter Umständen nicht die unterliegenden Gedanken- und Verhaltensmuster zu verändern. Auch wenn im Falle von MBSR in einigen Studien ein 6‑ bis 8‑wöchiges Programm mit 1,5 h wöchentlich und ca. 30-minütigem täglichen Üben untersucht wurde, so zeigte MBSR nur moderate Effektstärken [22] und zum Teil gab es auch inkonsistente Befunde [21].

Bei den Achtsamkeitsansätzen können, ähnlich wie bei der KVT, Nachteile dadurch entstehen, dass die Entdeckung tiefer liegender Ursachen, z. B. von Leistungsstreben, nicht Teil der Therapie sind und deshalb Erlebens- und Verhaltensänderungen nicht dauerhaft bestehen bleiben.

### Akzeptanz- und Commitment-Therapie (ACT)

In der Akzeptanz- und Commitment-Therapie [28] geht es darum, die psychische Flexibilität zu erhöhen. Dies passiert durch Achtsamkeit, Akzeptanz von negativen Gedanken und Reflexion von Werten. Einen wichtigen Pfeiler stellt zudem das engagierte Handeln (Commitment) dar, das auch aufrechterhalten bleiben sollte, wenn Herausforderungen auftreten, und das Selbst im Kontext von Disstress mitsamt allen Bewertungen, Gefühlen und Gedanken sowie Rollen und Erwartungen analysiert. Als weiterer Faktor kommt noch die „Defusion“ mit ins Spiel, eine Technik, um Abstand zwischen dem Selbst und Gefühlen zu schaffen. Vor allem die erhöhte psychische Flexibilität und die Fähigkeit, mit Rückschlägen gut umzugehen, können sehr hilfreich für Burnout-Patienten sein (vgl. [29]).

Die ACT hilft beispielsweise dabei, Schmerz und unangenehme Erlebnisse nicht mehr zu vermeiden, sondern sich mit ihnen zu konfrontieren. Dies kann zur Lösung der Probleme sinnvoll sein, jedoch auch als überfordernd erlebt werden. Da sich bei Burnout die Personen schon in einem Zustand der Erschöpfung befinden, ist hier die Frage, inwiefern diese Techniken bei einem hohen Grad an Burnout noch angewendet werden sollten. Ein weiterer zu beachtender Aspekt ist, dass möglicherweise selbst bei gelernter Akzeptanz in stressverursachenden Situationen die Wirkung der Stressoren im Sinne von physiologischen Stressreaktionen noch anhält, so wie beispielsweise bei Lärm, der als Stressor zwar bewusst ausgeblendet, also akzeptiert werden kann, dennoch aber andauernde physiologische Konsequenzen hat [30].

### Rational-Emotive-Therapie (RET)

Die Rational-Emotive-Therapie [31] fokussiert auf irrationale Denkmuster, wobei irrationale und negative Grundannahmen und daraus resultierende Konsequenzen analysiert werden. Dabei erfolgt auch Hilfestellung bei der Entwicklung eines Verständnisses dafür, wodurch der Burnout entstanden sein könnte.

Die konfrontative Art und der Fokus auf Selbstverantwortung in der RET könnten zum Teil jedoch auch, je nach Schweregrad des Burnouts, überfordernd sein. In einer Studie, die metaanalytisch die Effekte von RET untersuchte, betrug die Therapielänge in einem Rahmen von 2–10 Wochen 2‑mal wöchentlich 80–90 min. Die Ergebnisse zeigen hier große Effektstärken. Dabei ist zu erkennen, dass es sinnvoll sein könnte, insbesondere die Muster und Überzeugungen näher zu betrachten, die für die Entwicklung des Burnouts mitverantwortlich waren.

### Weitere mögliche Therapieansätze bei Burnout

Die in den von uns ausgewerteten Reviews bzw. Metaanalysen evaluierten Therapieansätze für Burnout können auf jeden Fall kurzfristig zu einer Symptombesserung führen. Ob sie aber nachhaltig und für das gesamte Spektrum von Burnout ausreichend geeignet sind, bleibt noch offen. Bei der Frage nach weiteren Therapieansätzen ist festzuhalten, dass sich Burnout ausschließlich durch beruflichen Stress entwickelt [32].

Arbeits- und berufsbezogenen Stressoren kann in der Therapie durch den Aufbau von Ressourcen begegnet werden [33]. Erste Studien bestätigen die Wirksamkeit von *ressourcenorientierter Therapie* bei Burnout [34]. Ressourcen sind zum Beispiel Optimismus, Kontrollerleben, Selbstakzeptanz, Vergebung und Sinn im Leben. Theoriebasiert ist davon auszugehen, dass diese Ressourcen wie Aufwärtsspiralen wirken und so die Gesundung unterstützen [35]. Dabei kann die erweiterte Aufmerksamkeit, die durch die Förderung der persönlichen Ressourcen entsteht, dazu führen, dass gute Lösungen für die Herausforderungen des Alltags gefunden werden. Gerade in der Behandlung von Burnout ist es ratsam, sich auf die Arbeit an den veränderbaren Ressourcen zu fokussieren.

#### Optimismus.

Als eine generische Persönlichkeitsvariable gibt Optimismus den Grad an, zu dem eine Person positive und erwartungsvolle Zukunftsansichten hegt [36, 37]. Optimismus geht dabei mit höheren physiologischem und psychologischem Wohlbefinden einher [38] und gilt als protektiver Faktor für Burnout [39]. Genauso, wie zum Beispiel Hoffnung durch positive Emotionen im Rahmen einer Meditation aufgebaut werden kann [40], könnte auch Optimismus adressiert werden. Auch Interventionen wie kognitive Umbewertung (Reappraisal) können Optimismus stärken (siehe [38, S. 886] für eine Diskussion über die Veränderung). Eine Ressource wie Optimismus unterstützt generell beim Erreichen von Zielen [41, 42], was wiederum Selbstakzeptanz und das Kontrollerleben stärkt.

#### Kontrolle.

Die wahrgenommene Kontrolle ist einer der bekanntesten Schutzfaktoren gegen Belastungsreaktionen [43]. Das Arbeitsstressmodell von Karasek [32] besagt, je höher die Kontrolle ist, desto geringer sind die Effekte von Stressoren auf Belastungsreaktionen [32]. Wahrgenommene Kontrolle, die unabhängig von tatsächlicher Kontrolle ist, kann zum Beispiel im therapeutischen Rahmen dadurch gefördert werden, dass unterschiedliche Handlungsoptionen erarbeitet werden. Dabei ist es von Vorteil, wenn die Dinge von innen heraus als veränderbar wahrgenommen werden, weil sich dies auf die Zufriedenheit, die Leistung und die Gesundheit auswirken kann [44, 45].

#### Selbstwirksamkeit.

Die Selbstwirksamkeit, der Glaube an die Erfolgswahrscheinlichkeit des eigenen Handelns, ist eng mit Kontrolle verbunden. Das Konzept der Selbstwirksamkeit [46] hat sich ebenfalls als Ressource im Zusammenhang mit Belastungsreaktionen gezeigt und erscheint somit interessant in der Behandlung von Burnout.

#### Selbstakzeptanz.

Als zentrale Variable für psychologisches Wohlbefinden und persönliche Entwicklung gilt die Selbstakzeptanz. Sie ist definiert als eine positive Haltung sich selbst gegenüber [47]. Personen, die über eine hohe Selbstakzeptanz verfügen, akzeptieren sowohl ihre positiven als auch ihre negativen Aspekte. Im Zusammenhang mit Burnout ist Selbstakzeptanz als Mediator erforscht. Niedrige Selbstakzeptanz erklärt beispielsweise den Effekt von Perfektionismus auf Burnout [48]. Die Selbstakzeptanz ist im Laufe des Lebens veränderlich [49, 50] und kann auch durch gezielte therapeutische Interaktionen verändert werden [51]. Eine Methode ist es, in einem gezielten Dialog die mit der Selbstakzeptanz verwandten Konzepte, wie Furcht vor Misserfolgen, Wertlosigkeit und Perfektionismus, therapeutisch zu bearbeiten und dadurch die Selbstakzeptanz zu erhöhen, was auch zu einer Verbesserung des „Sich-seiner-selbst-bewusst-Seins“ führt [51].

### Aspekte, die bei der Burnout-Therapie zu beachten sind

Es besteht die Gefahr, dass sich hinter einer schwereren Ausprägung von Burnout eine „larvierte“ psychische Erkrankung verbirgt und sich diese nicht durch Techniken, die nur im „Hier und Jetzt“ ansetzen, behandeln lässt. Wird allerdings umgekehrt im Rahmen einer Richtlinientherapie bei einer „klassischen psychischen Störung“, wie einer Depression, nicht ausreichend mit einbezogen, dass der Depression eine Burnout-Entwicklung vorausging oder diese begleitend besteht, kann dies ebenfalls dazu führen, dass der Therapieerfolg nicht ausreichend nachhaltig ist.

Was aber bei allen Möglichkeiten, Burnout-Betroffene therapeutisch zu unterstützen, nicht aus dem Blick geraten darf, ist, dass Burnout per definitionem im Wesentlichen durch Arbeitsbedingungen ausgelöst wird [2]. Hier sind Arbeitgeber gefragt, auf verhältnispräventiver Ebene aktiv zu werden. Um Burnout zu adressieren, sollte generell der Blick auf den Arbeitskontext, berufliche Stressoren, präventive Faktoren und spezifische Ressourcen gelegt werden. In der Therapie von Erkrankungen, die aus einer Burnout-Entwicklung resultieren, können die in diesem Umbrella-Review vorgestellten Methoden ebenso wie die tiefer gehenden und den Arbeitskontext im Besonderen adressierenden Ansätze eine Ergänzung in der Therapie darstellen, die den Spezifika des Burnouts Rechnung tragen.

Bei der Frage nach geeigneten Methoden sollte aber auch den unterschiedlichen Settings im Gesundheitswesen, in denen Burnout-Patienten vorstellig werden, Rechnung getragen werden. Zur Burnout-Behandlung im Sinne der Prävention psychischer Folgeerkrankungen (Sekundärprävention) können die im Artikel vorgestellten Ansätze eine Hilfestellung sein, sowohl für Hausärzte, die in der Primärversorgung meist die ersten Ansprechpartner für Betroffene sind, als auch für Psychotherapeuten, die in der Probatorik oder Kurzzeittherapie Burnout-Betroffenen zielgerichtet helfen möchten, um schwerwiegendere Folgen abzuwenden.

## Fazit

Die im Umbrella-Review vorgestellten Therapieformen kognitive Verhaltenstherapie, Achtsamkeitstraining, Akzeptanz- und Commitment-Therapie sowie Rational-Emotive-Therapie sind hauptsächlich darauf ausgerichtet, die Symptome von Burnout zeitnah effektiv zu lindern. Dies erscheint in Anbetracht der Tatsache, dass Burnout keine Erkrankung im eigentlichen Sinn darstellt und derzeit eher als „Vorstufe“ zu einer psychischen Erkrankung gesehen wird, nachvollziehbar und sinnvoll. Doch selbst wenn es sich bei Burnout „noch“ um einen nicht krankheitswertigen Zustand handelt, stecken meist tiefer gehende Ursachen hinter seiner Entwicklung. Und auch wenn durch die bislang evaluierten Techniken kurzfristig eine Symptomentlastung erreicht werden kann, besteht bei den Betroffenen das Risiko, im weiteren Berufsleben wieder in eine ähnliche Situation zu geraten, da sie sich nicht mit den tiefer liegenden intraindividuellen Ursachen ihres Bur-outs auseinandergesetzt haben. Neben den genannten Therapieformen gibt es verschiedene, in der Zukunft noch weiter zu erforschende Ansatzmöglichkeiten, um die Burnout-Behandlung noch weiter zu verbessern. Diese Ansätze, die sich aus dem spezifischen Arbeitskontext von Burnout ableiten lassen und die die Burnout-Behandlung potenziell vertiefen und nachhaltiger gestalten könnten, zu Techniken weiterzuentwickeln, in Methoden einzubetten und auf ihre Wirksamkeit zu überprüfen, sollte Gegenstand weiterer Burnout-Forschung sein.

## Supplementary Information

Prisma Study Protocol
