# Supplementary material for: Psychotherapeutic burnout interventions—an umbrella review
Source: Bundesgesundheitsblatt Gesundheitsforschung Gesundheitsschutz. 2024 Oct 15;67(11):1279–87. [Article in German] doi: 10.1007/s00103-024-03961-y (PMC11549179; doi:10.1007/s00103-024-03961-y)
Supplement: Supplementary file 1 — Prisma Study Protocol [file 103_2024_3961_MOESM1_ESM.pdf]

Systematic Review

German Title: Psychotherapeutische Interventionen bei Burnout – Ein Umbrella-Review und Impulse für die Therapie

English Title: Psychotherapeutic Burnout Interventions – an umbrella review and outlook for treatment options

Authors: Kern, S., Jerg-Bretzke, L., Beschoner, P.

BRIEF SUMMARY
According to studies, many people frequently suffer from work stress and burnout (Alarcon, 2011). The impetus for our umbrella review came from the need to better understand which therapies work well already and to take a look in what might be missing from the picture. This article will provide an umbrella review over existing studies which therapies counteract the costly consequences of burnout and show specific ways to address burnout in therapeutic relationships. Systematic literature search of studies in PubMed and Google Scholar. Search terms were „burnout“, „therapy“, combined with „systematic review“, „meta-analysis.“ So far, in the umbrella review cognitive behavioral therapy, acceptance and commitment-based therapy and mindfulness training were shown to be most effective in reducing burnout. However, we suggest that some other resources could be overlooked, with also could be useful in the treatment of burnout. Consequently, the study presentation makes an important contribution to the treatment of burnout, tailored psychotherapeutic intervention and secondary prevention strategies.

REVIEW TEAMMEMBERS/RESEARCHERS
Sarah Kern Psychosomatic Medicine and Psychotherapy, Ulm University Medical Center, Ulm, Germany
Lucia Jerg-Bretzke, Department of Psychosomatic Medicine and Psychotherapy, Ulm University Medical Center, Ulm, Germany
Petra Beschoner, Department of Psychosomatic Medicine and Psychotherapy, Ulm University Medical Center, Ulm, Germany

CONTACT
Sarah Kern, Department of Psychology, Stuttgart, IB Hochschule Stuttgart, Germany
Sarah.kern@ib-hochschule.de;

ORGANISATIONAL AFFILICATION
Department of Psychosomatic Medicine and Psychotherapy, Ulm University Medical Center, Ulm, Germany
https://www.uniklinik-ulm.de/psychosomatische-medizin-und-psychotherapie.html

REVIEW QUESTIONS
The review questions referred to systematically identify and synthesize the available literature on therapy and burnout

SEARCH STRATEGY
The systematic literature search was carried out in the period from 01.02. to 03.05.2024 in compliance with the PRISMA statements (Moher, Liberati, Tetzlaff, Altman, & Group, 2009) via the databases Pubmed and Google Scholar for the years 2010 - 2024 in order to obtain the most recent publications, if possible.

To ensure this, the terms "burnout" and "emotional exhaustion" were used in the searches. These were combined with terms for the targeted type of anaylsis “systematic review” and “meta-analysis.” As a third aspect, the concept intervention was included. Due to different spellings and types the following key words were chosen and combined for the analysis: "therapy," "intervention" and "individual intervention." The terms were linked with the Boolean operators "and" and "or" respectively. In addition, in Pub-Med the check boxes for “meta-analysis” and “systematic review” were activated. A second, analogous search was conducted with the first two word groups. The terms had to be included either in the title or abstract. The results were stored separately for each database

and fed into Citavi Version 6.

SELECTION CRITERIA

Area	Inclusion	Exclusion
Participants/Population	- All sexes<br>- Working age	- Athletes
Interventions	Therapy-based interventions<br>- ACT<br>- MBSR<br>- RET<br>- ACT	- Workplace intervention<br>- Organizational strategies<br>- Art-based therapy interventions<br>- Intervention program (no therapy)<br>- Factors associated with burnout<br>- Educational style interventions<br>- Prevalence of burnout<br>- Group-based training /interventions<br>- Sport-Therapy<br>- Sports, Exercise Therapy<br>- General Recommendations<br>- Comparison of individual and structural treatments<br>- Meditation
Comparator(s)/control	No selection criteria	
Outcomes	Burnout (MBI)	
Publication date	2010-2024 (current)	
Publication language	English	
Publication format	No restrictions	

Exclusion:
In addition, every paper needed to be a systematic review or a meta-analysis in itself

CONTEXT
According to studies, many people frequently suffer from work stress and burnout. This article will provide an umbrella review over existing studies which therapies counteract the costly consequences of burnout and show specific ways to address burnout in therapeutic relationships. So far, in the umbrella review cognitive behavioral therapy, acceptance and commitment-based therapy and mindfulness training were shown to be most effective in reducing burnout. However, we suggest that some other resources could be overlooked, with also could be useful in the treatment of burnout.

REVIEW PROCESS
The systematic review was performed in accordance with the PRISMA-ScR declaration (Tricco et al., 2018)

STUDY SELECTION
Duplicates were removed using Citavi. The remaining publications were checked for content relevance by means of the titles, then the abstracts and finally the content. If the respective publications did not show any relevance, they were excluded. Subsequently, bibliographical references from the works already found were identified and checked using the reverse search (Skelton, 1973). The forward search (Garfield, 1964) was used to find all recent publications citing the reference articles from the available, relevant articles via the specialized subject database Web of Science and via Google Scholar.

Unclear cases at the level of title and abstract screening were discussed between the authors (SK, PB, J-B). Subsequently, the full texts of the relevant studies were inspected to determine which studies meet the inclusion criteria. The full text screening was performed in duplicate by two independent reviewers (SK and PB). Any differences of opinion were settled by discussion or by convening a third reviewer (J-B).

DATA EXTRACTION

Relevant information for each included study was reviewed by two reviewers (SK, PB), which worked independently of each other using a questionnaire, developed for this review.
The following information was collected:
- Study (authors, year)
- Sample size (number participants)
- outcome parameters (instruments for recording burnout)
- Main results regarding the mentioned outcome parameters and possible interrelationships

Discrepancies in the data collection were clarifyed by discussion or by consulting a third reviewer (J-B).

DATA ANALYSIS

No assessment of the risk of bias has been made.

Data Synthesis

The included studies were summarized narratively. No pooled analysis of the quantitative results. The publications were summarized in tabular form with sample size and results

TYPE AND METHOD OF REVIEW

Umbrella review of systematic reviews and meta-analysis; intervention; narrative summary

KEYWORDS

Burnout, therapy, umbrella review, treatment, resources

START DATE:	2024-February
COMPLETE DATE:	2024-May
LANGUAGE:	German
COUNTRY:	Germany
FUNDING SOURCES:	No third-party funding

CONFLICTS OF INTEREST:

SK, LJB and PB have no conflicts of interest.

CURRENT REVIEW STATUS

Preliminary searches: completed
Piloting of the study selection process: completed
Formal screening of search results against eligibility criteria: completed
Data extraction: completed
Risk of bias (quality) assessment: n.a. (umbrella review)
Data analysis: completed

The protocol for our umbrella review was roughly sketched before the review was prepared, but was not published. At the suggestion of the reviewer during the publication process, the detailed protocol was prepared and published.

REFERENCES

Alarcon, G. M. (2011). A meta-analysis of burnout with job demands, resources, and attitudes. *Journal of Vocational Behavior, 79*, 549-562. doi:10.1016/j.jvb.2011.03.007

Garfield, E. (1964). Citation Indexing: A Natural Science Literature Retrieval System for the Social Sciences. *American Behavioral Scientist, 7*(10), 58-61. doi:10.1177/000276426400701017

Moher, D., Liberati, A., Tetzlaff, J., Altman, D. G., & Group, P. (2009). Preferred reporting items for systematic reviews and meta-analyses: the PRISMA statement. *PLoS Med, 6*(7), e1000097. doi:10.1371/journal.pmed.1000097

Skelton, B. (1973). Scientists and Social Scientists as Information Users: a comparison of results of science user studies with the investigation into information requirements of the social sciences. *Journal of librarianship, 5*(2), 138-156. doi:10.1177/096100067300500205

Tricco, A. C., Lillie, E., Zarin, W., O'Brien, K. K., Colquhoun, H., Levac, D., . . . Straus, S. E. (2018). PRISMA Extension for Scoping Reviews (PRISMA-ScR): Checklist and Explanation. *Ann Intern Med, 169*(7), 467-473. doi:10.7326/m18-0850
